# 4-(3-Iodo­phen­yl)-1-(2-oxoindolin-3-yl­idene)thio­semicarbazide

**DOI:** 10.1107/S1600536810021938

**Published:** 2010-06-16

**Authors:** Humayun Pervez, Muhammad Yaqub, Muhammad Ramzan, M. Nawaz Tahir

**Affiliations:** a Department of Chemistry, Bahauddin Zakariya University, Multan 60800, Pakistan; b Department of Physics, University of Sargodha, Sargodha, Pakistan

## Abstract

In the title compound, C_15_H_11_IN_4_OS, intra­molecular N—H⋯N, N—H⋯O and C—H⋯S inter­actions generate one *S*(5) and two *S*(6) ring motifs. In the crystal, mol­ecules form centrosymmetric dimers *via* pairs of N—H⋯O inter­actions, generating *R*_2_^2^(8) ring motifs. In addition a short inter­molecular I⋯S contact of 3.352 (3) Å is observed.

## Related literature

For the preparation of biologically important *N* ^4^-aryl-substituted isatin-3-thio­semicarbazones, see: Pervez *et al.* (2007 ▶, 2008 ▶, 2010*a* ▶). For a related structure, see: Pervez *et al.* (2010*b* ▶). For graph-set notation, see: Bernstein *et al.* (1995 ▶).
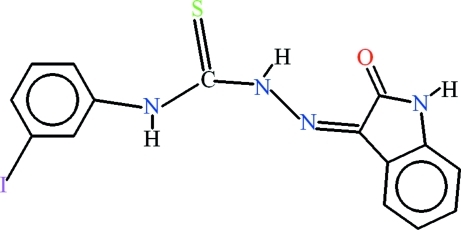


## Experimental

### 

#### Crystal data

C_15_H_11_IN_4_OS
*M* *_r_* = 422.24
Monoclinic, 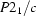

*a* = 5.7620 (3) Å
*b* = 16.7989 (11) Å
*c* = 16.152 (1) Å
β = 100.153 (4)°
*V* = 1538.96 (16) Å^3^
*Z* = 4
Mo *K*α radiation
μ = 2.22 mm^−1^
*T* = 296 K
0.32 × 0.14 × 0.12 mm

#### Data collection

Bruker Kappa APEXII CCD diffractometer
Absorption correction: multi-scan (*SADABS*; Bruker, 2005 ▶) *T*_min_ = 0.742, *T*_max_ = 0.752
11310 measured reflections
2778 independent reflections
1677 reflections with *I* > 2σ(*I*)
*R*_int_ = 0.022

#### Refinement

*R*[*F* ^2^ > 2σ(*F* ^2^)] = 0.055
*wR*(*F* ^2^) = 0.157
*S* = 1.01
2778 reflections
199 parameters
H-atom parameters constrained
Δρ_max_ = 2.76 e Å^−3^
Δρ_min_ = −0.48 e Å^−3^

### 

Data collection: *APEX2* (Bruker, 2007 ▶); cell refinement: *SAINT* (Bruker, 2007 ▶); data reduction: *SAINT*; program(s) used to solve structure: *SHELXS97* (Sheldrick, 2008 ▶); program(s) used to refine structure: *SHELXL97* (Sheldrick, 2008 ▶); molecular graphics: *ORTEP-3 for Windows* (Farrugia, 1997 ▶) and *PLATON* (Spek, 2009 ▶); software used to prepare material for publication: *WinGX* (Farrugia, 1999 ▶) and *PLATON*.

## Supplementary Material

Crystal structure: contains datablocks global, I. DOI: 10.1107/S1600536810021938/gk2281sup1.cif

Structure factors: contains datablocks I. DOI: 10.1107/S1600536810021938/gk2281Isup2.hkl

Additional supplementary materials:  crystallographic information; 3D view; checkCIF report

## Figures and Tables

**Table 1:** Hydrogen-bond geometry (Å, °)

*D*—H⋯*A*	*D*—H	H⋯*A*	*D*⋯*A*	*D*—H⋯*A*
N1—H1⋯O1^i^	0.86	2.09	2.939 (9)	169
N3—H3*A*⋯O1	0.86	2.07	2.761 (9)	137
N4—H4*A*⋯N2	0.86	2.18	2.618 (9)	111
C15—H15⋯S1	0.93	2.51	3.183 (10)	129

Symmetry code: (i) 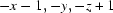.

**Fig. 1.**
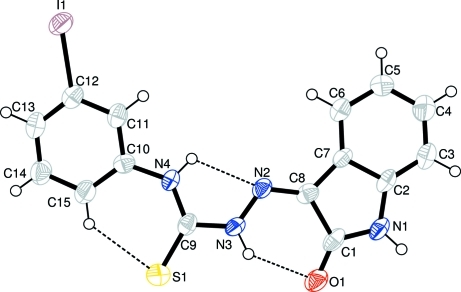
View of the title molecule with the atom numbering scheme. The displacement ellipsoids are drawn at the 50% probability level. H-atoms are shown by small circles of arbitrary radii. The dotted lines indicate intramolecular hydrogen bonds.

**Fig. 2.**
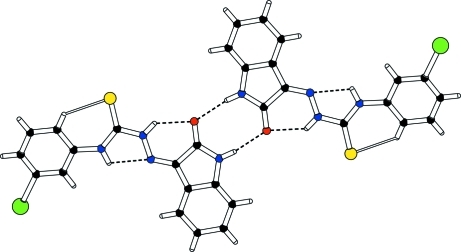
The partial packing (PLATON; Spek, 2009) which shows that molecules form dimers.

## supplementary crystallographic information

### Comment

As a part of our drug discovery program, we very recently reported the synthesis and biological evaluation of a number of *N*^4^-aryl substituted isatins-thiosemicarbazones (Pervez *et al.*, 2007, 2008, 2010*a*). In continuation of the same, we report herein the structure and synthesis of the title compound (I, Fig. 1).

The crystal structure of (II) *i.e.* 4-(3-methoxyphenyl)-1-(2-oxoindolin-3-ylidene)thiosemicarbazides has been published (Pervez *et al.*, 2010*b*). The title compound differs from (II) due to the presence of iodo instead of methoxy function at position-3 of the phenyl ring substituted at N^4^ of the thiosemicarbazone moiety.

In (I) the 2-oxoindolin A (C1–C8/N1/O1), thiosemicarbazide B (N2/N3/C9/S1/N4) and phenyl ring of 2-ethylphenyl C (C10—C16) are planar with r. m. s. deviations of 0.0086, 0.0029 and 0.0414 Å, respectively. The dihedral angle between A/B, A/C and B/C is 4.65 (41)°, 11.89 (41)° and 13.37 (37)°, respectively. Due to intramolecular H-bondings (Table 1, Fig. 1), one S(5) and two S(6) (Bernstein *et al.*, 1995) ring motifs are formed. The molecules are dimerized (Fig. 2) due to intermolecular H-bonding of N—H···O type with *R*_2_^2^(8) ring motifs.

### Experimental

To a hot solution of isatin (0.74 g, 5.0 mmol) in ethanol (10 ml) containing a few drops of glacial acetic acid was added 4-(3-iodophenyl)thiosemicarbazide (1.47 g, 5.0 mmol) dissolved in ethanol (10 ml) under stirring. The reaction mixture was then heated under reflux for 2 h. The yellow crystalline solid formed during heating was collected by suction filtration. Thorough washing with hot ethanol followed by ether afforded the target compound (I) in pure form (1.77 g, 84%), m. p. 503 K (*d*). The single crystals of (I) were grown in acetone-ethanol (1:4) by diffusion method at room temperature.

### Refinement

All H-atoms were positioned geometrically (N–H = 0.86 Å, C–H = 0.93 Å) and refined as riding with *U*_iso_(H) = *xU*_eq_(C, N), where *x* = 1.2 for all H-atoms. A residual peak of 2.76 e/Å^3^ exists at a distance of 1.48 and 1.95 Å from C14 and C13, respectively.

### Crystal data

**Table:**

C_15_H_11_IN_4_OS	*F*(000) = 824
*M**_r_* = 422.24	*D*_x_ = 1.822 Mg m^−^^3^
Monoclinic, *P*2_1_/*c*	Mo *K*α radiation, λ = 0.71073 Å
Hall symbol: -P 2ybc	Cell parameters from 1677 reflections
*a* = 5.7620 (3) Å	θ = 2.7–25.3°
*b* = 16.7989 (11) Å	µ = 2.22 mm^−^^1^
*c* = 16.152 (1) Å	*T* = 296 K
β = 100.153 (4)°	Needle, yellow
*V* = 1538.96 (16) Å^3^	0.32 × 0.14 × 0.12 mm
*Z* = 4	

### Data collection

**Table:**

Bruker Kappa APEXII CCD diffractometer	2778 independent reflections
Radiation source: fine-focus sealed tube	1677 reflections with *I* > 2σ(*I*)
graphite	*R*_int_ = 0.022
Detector resolution: 8.10 pixels mm^-1^	θ_max_ = 25.3°, θ_min_ = 2.7°
ω scans	*h* = −6→6
Absorption correction: multi-scan (*SADABS*; Bruker, 2005)	*k* = −20→20
*T*_min_ = 0.742, *T*_max_ = 0.752	*l* = −16→19
11310 measured reflections	

### Refinement

**Table:**

Refinement on *F*^2^	Primary atom site location: structure-invariant direct methods
Least-squares matrix: full	Secondary atom site location: difference Fourier map
*R*[*F*^2^ > 2σ(*F*^2^)] = 0.055	Hydrogen site location: inferred from neighbouring sites
*wR*(*F*^2^) = 0.157	H-atom parameters constrained
*S* = 1.01	*w* = 1/[σ^2^(*F*_o_^2^) + (0.0776*P*)^2^ + 1.1429*P*] where *P* = (*F*_o_^2^ + 2*F*_c_^2^)/3
2778 reflections	(Δ/σ)_max_ < 0.001
199 parameters	Δρ_max_ = 2.76 e Å^−^^3^
0 restraints	Δρ_min_ = −0.47 e Å^−^^3^

### Special details

**Table:**

Geometry. Bond distances, angles *etc*. have been calculated using the rounded fractional coordinates. All su's are estimated from the variances of the (full) variance-covariance matrix. The cell e.s.d.'s are taken into account in the estimation of distances, angles and torsion angles
Refinement. Refinement of *F*^2^ against ALL reflections. The weighted *R*-factor *wR* and goodness of fit *S* are based on *F*^2^, conventional *R*-factors *R* are based on *F*, with *F* set to zero for negative *F*^2^. The threshold expression of *F*^2^ > σ(*F*^2^) is used only for calculating *R*-factors(gt) *etc*. and is not relevant to the choice of reflections for refinement. *R*-factors based on *F*^2^ are statistically about twice as large as those based on *F*, and *R*- factors based on ALL data will be even larger.

### Fractional atomic coordinates and isotropic or equivalent isotropic displacement parameters (Å^2^)

**Table:**

	*x*	*y*	*z*	*U*_iso_*/*U*_eq_	
I1	1.07185 (10)	0.31162 (4)	0.23986 (4)	0.0483 (3)	
S1	0.4332 (4)	0.20616 (18)	0.59942 (16)	0.0586 (10)	
O1	−0.2092 (9)	0.0552 (4)	0.5161 (4)	0.045 (2)	
N1	−0.4370 (12)	0.0106 (4)	0.3922 (4)	0.040 (3)	
N2	0.0812 (11)	0.1226 (4)	0.3935 (4)	0.034 (2)	
N3	0.1620 (11)	0.1406 (4)	0.4736 (4)	0.037 (3)	
N4	0.4525 (10)	0.2139 (4)	0.4329 (4)	0.034 (2)	
C1	−0.2557 (14)	0.0481 (5)	0.4378 (6)	0.039 (3)	
C2	−0.4343 (14)	0.0140 (5)	0.3070 (6)	0.038 (3)	
C3	−0.5883 (15)	−0.0147 (6)	0.2406 (6)	0.050 (3)	
C4	−0.5463 (17)	−0.0022 (6)	0.1609 (7)	0.058 (4)	
C5	−0.3504 (17)	0.0387 (6)	0.1472 (6)	0.055 (4)	
C6	−0.1928 (16)	0.0702 (6)	0.2141 (6)	0.049 (3)	
C7	−0.2329 (13)	0.0575 (5)	0.2942 (5)	0.035 (3)	
C8	−0.1107 (13)	0.0803 (5)	0.3755 (5)	0.036 (3)	
C9	0.3534 (13)	0.1884 (5)	0.4975 (5)	0.036 (3)	
C10	0.6498 (14)	0.2638 (5)	0.4318 (5)	0.035 (3)	
C11	0.7303 (13)	0.2695 (5)	0.3565 (6)	0.038 (3)	
C12	0.9304 (14)	0.3131 (5)	0.3511 (5)	0.038 (3)	
C13	1.0440 (15)	0.3535 (5)	0.4199 (6)	0.044 (3)	
C14	0.9691 (16)	0.3505 (6)	0.4947 (6)	0.050 (3)	
C15	0.7723 (16)	0.3050 (6)	0.5021 (6)	0.048 (3)	
H1	−0.54474	−0.01324	0.41344	0.0478*	
H3	−0.72140	−0.04269	0.24913	0.0602*	
H3A	0.09085	0.12147	0.51176	0.0443*	
H4	−0.65213	−0.02171	0.11525	0.0694*	
H4A	0.38484	0.19719	0.38428	0.0404*	
H5	−0.32307	0.04539	0.09258	0.0658*	
H6	−0.06263	0.09934	0.20473	0.0588*	
H11	0.64938	0.24387	0.30899	0.0460*	
H13	1.17608	0.38384	0.41514	0.0529*	
H14	1.04853	0.37862	0.54072	0.0598*	
H15	0.72130	0.30179	0.55351	0.0580*	

### Atomic displacement parameters (Å^2^)

**Table:**

	*U*^11^	*U*^22^	*U*^33^	*U*^12^	*U*^13^	*U*^23^
I1	0.0373 (4)	0.0617 (5)	0.0487 (4)	0.0009 (3)	0.0153 (3)	0.0093 (3)
S1	0.0525 (15)	0.083 (2)	0.0409 (14)	−0.0148 (14)	0.0096 (11)	0.0018 (14)
O1	0.038 (3)	0.052 (4)	0.050 (4)	−0.003 (3)	0.021 (3)	−0.001 (3)
N1	0.034 (4)	0.039 (4)	0.052 (5)	−0.007 (3)	0.020 (3)	0.000 (4)
N2	0.026 (4)	0.041 (4)	0.038 (4)	−0.002 (3)	0.011 (3)	0.002 (3)
N3	0.032 (4)	0.048 (5)	0.034 (4)	−0.006 (3)	0.012 (3)	0.005 (4)
N4	0.026 (4)	0.037 (4)	0.040 (4)	−0.005 (3)	0.009 (3)	0.001 (3)
C1	0.030 (5)	0.033 (5)	0.058 (7)	0.004 (4)	0.023 (4)	0.005 (5)
C2	0.027 (4)	0.036 (5)	0.054 (6)	−0.002 (4)	0.013 (4)	0.008 (5)
C3	0.039 (5)	0.048 (6)	0.063 (7)	−0.014 (4)	0.010 (5)	0.002 (5)
C4	0.051 (6)	0.063 (7)	0.058 (7)	−0.013 (5)	0.006 (5)	−0.001 (6)
C5	0.063 (6)	0.063 (7)	0.039 (6)	−0.016 (5)	0.011 (5)	0.001 (5)
C6	0.049 (5)	0.049 (6)	0.051 (6)	−0.015 (5)	0.016 (5)	0.000 (5)
C7	0.036 (4)	0.032 (5)	0.040 (5)	−0.005 (4)	0.016 (4)	0.002 (4)
C8	0.027 (4)	0.044 (6)	0.039 (5)	0.006 (4)	0.010 (4)	0.007 (4)
C9	0.026 (4)	0.037 (5)	0.045 (5)	0.001 (4)	0.007 (4)	0.006 (4)
C10	0.035 (5)	0.036 (5)	0.036 (5)	0.005 (4)	0.010 (4)	0.003 (4)
C11	0.027 (4)	0.036 (5)	0.051 (6)	0.005 (4)	0.004 (4)	0.005 (5)
C12	0.029 (4)	0.035 (5)	0.049 (6)	0.009 (4)	0.008 (4)	0.010 (5)
C13	0.032 (5)	0.039 (6)	0.061 (7)	−0.004 (4)	0.005 (4)	0.003 (5)
C14	0.047 (6)	0.048 (6)	0.057 (6)	−0.012 (5)	0.014 (5)	−0.012 (5)
C15	0.040 (5)	0.055 (6)	0.052 (6)	−0.008 (5)	0.014 (4)	−0.005 (5)

### Geometric parameters (Å, °)

**Table:**

I1—C12	2.100 (8)	C5—C6	1.388 (14)
S1—C9	1.656 (8)	C6—C7	1.371 (12)
O1—C1	1.251 (11)	C7—C8	1.429 (11)
N1—C1	1.326 (11)	C10—C15	1.409 (13)
N1—C2	1.380 (11)	C10—C11	1.379 (12)
N2—N3	1.330 (9)	C11—C12	1.382 (11)
N2—C8	1.303 (10)	C12—C13	1.367 (12)
N3—C9	1.364 (10)	C13—C14	1.354 (13)
N4—C9	1.345 (10)	C14—C15	1.390 (14)
N4—C10	1.415 (10)	C3—H3	0.9300
N1—H1	0.8600	C4—H4	0.9300
N3—H3A	0.8600	C5—H5	0.9300
N4—H4A	0.8600	C6—H6	0.9300
C1—C8	1.517 (12)	C11—H11	0.9300
C2—C3	1.355 (13)	C13—H13	0.9300
C2—C7	1.417 (11)	C14—H14	0.9300
C3—C4	1.367 (14)	C15—H15	0.9300
C4—C5	1.372 (14)		
			
I1···S1^i^	3.352 (3)	C9···O1^iv^	3.344 (10)
I1···S1^ii^	3.979 (3)	C10···C8^iv^	3.560 (12)
I1···H3^iii^	3.2000	C11···C8^iv^	3.307 (12)
I1···H4A^iv^	3.3000	C11···N2^iv^	3.180 (11)
S1···C15	3.183 (10)	C12···N2^iv^	3.355 (11)
S1···I1^v^	3.352 (3)	C13···N4^iv^	3.303 (11)
S1···I1^vi^	3.979 (3)	C13···C9^iv^	3.414 (12)
S1···H15	2.5100	C14···C9^iv^	3.505 (13)
O1···N2	3.028 (9)	C15···S1	3.183 (10)
O1···N3	2.761 (9)	C1···H3A	2.4700
O1···C9^vii^	3.344 (10)	C1···H1^ix^	2.9000
O1···C1^viii^	3.168 (10)	C1···H3A^viii^	3.0700
O1···O1^viii^	3.157 (8)	C5···H13^x^	3.0200
O1···N1^ix^	2.939 (9)	C9···H15	2.8700
O1···C8^viii^	3.241 (10)	C12···H3^iii^	3.0400
O1···H3A	2.0700	C13···H4^iii^	3.0600
O1···H1^ix^	2.0900	C14···H5^xi^	3.0500
N1···O1^ix^	2.939 (9)	C15···H5^xi^	3.0100
N2···O1	3.028 (9)	H1···O1^ix^	2.0900
N2···N4	2.618 (9)	H1···C1^ix^	2.9000
N2···C11^vii^	3.180 (11)	H3···I1^xii^	3.2000
N2···C12^vii^	3.355 (11)	H3···C12^xii^	3.0400
N3···O1	2.761 (9)	H3A···O1	2.0700
N4···C1^iv^	3.247 (11)	H3A···C1	2.4700
N4···N2	2.618 (9)	H3A···C1^viii^	3.0700
N4···C13^vii^	3.303 (11)	H4···C13^xii^	3.0600
N2···H4A	2.1800	H4A···I1^vii^	3.3000
C1···N4^vii^	3.247 (11)	H4A···N2	2.1800
C1···C9^vii^	3.511 (12)	H4A···H11	2.2500
C1···O1^viii^	3.168 (10)	H5···C14^xiii^	3.0500
C8···O1^viii^	3.241 (10)	H5···C15^xiii^	3.0100
C8···C10^vii^	3.560 (12)	H11···H4A	2.2500
C8···C11^vii^	3.307 (12)	H13···C5^xiv^	3.0200
C9···C14^vii^	3.505 (13)	H15···S1	2.5100
C9···C1^iv^	3.511 (12)	H15···C9	2.8700
C9···C13^vii^	3.414 (12)		
			
C1—N1—C2	112.8 (7)	S1—C9—N4	129.2 (6)
N3—N2—C8	118.6 (7)	C11—C10—C15	118.6 (8)
N2—N3—C9	122.4 (6)	N4—C10—C15	124.8 (7)
C9—N4—C10	130.6 (7)	N4—C10—C11	116.6 (7)
C2—N1—H1	124.00	C10—C11—C12	120.4 (8)
C1—N1—H1	124.00	I1—C12—C13	119.9 (6)
N2—N3—H3A	119.00	I1—C12—C11	120.1 (6)
C9—N3—H3A	119.00	C11—C12—C13	119.8 (8)
C10—N4—H4A	115.00	C12—C13—C14	121.7 (8)
C9—N4—H4A	115.00	C13—C14—C15	119.4 (9)
O1—C1—N1	127.9 (8)	C10—C15—C14	120.1 (8)
N1—C1—C8	105.9 (7)	C2—C3—H3	120.00
O1—C1—C8	126.3 (7)	C4—C3—H3	120.00
C3—C2—C7	120.5 (8)	C3—C4—H4	119.00
N1—C2—C3	130.8 (8)	C5—C4—H4	119.00
N1—C2—C7	108.7 (7)	C4—C5—H5	120.00
C2—C3—C4	119.4 (9)	C6—C5—H5	120.00
C3—C4—C5	121.1 (10)	C5—C6—H6	121.00
C4—C5—C6	120.6 (9)	C7—C6—H6	121.00
C5—C6—C7	118.7 (9)	C10—C11—H11	120.00
C6—C7—C8	133.5 (8)	C12—C11—H11	120.00
C2—C7—C6	119.8 (8)	C12—C13—H13	119.00
C2—C7—C8	106.7 (7)	C14—C13—H13	119.00
N2—C8—C1	126.3 (7)	C13—C14—H14	120.00
C1—C8—C7	105.9 (7)	C15—C14—H14	120.00
N2—C8—C7	127.8 (7)	C10—C15—H15	120.00
S1—C9—N3	117.2 (6)	C14—C15—H15	120.00
N3—C9—N4	113.6 (7)		
			
C1—N1—C2—C3	−178.2 (9)	C3—C2—C7—C6	−0.4 (13)
C2—N1—C1—O1	−179.7 (8)	C3—C2—C7—C8	179.0 (8)
C2—N1—C1—C8	−0.6 (9)	C2—C3—C4—C5	0.2 (15)
C1—N1—C2—C7	0.2 (10)	C3—C4—C5—C6	−1.7 (16)
N3—N2—C8—C7	176.9 (8)	C4—C5—C6—C7	2.0 (15)
C8—N2—N3—C9	−176.1 (7)	C5—C6—C7—C8	179.8 (9)
N3—N2—C8—C1	−0.3 (12)	C5—C6—C7—C2	−1.0 (13)
N2—N3—C9—S1	179.6 (6)	C2—C7—C8—N2	−178.4 (8)
N2—N3—C9—N4	−1.0 (11)	C6—C7—C8—N2	0.9 (16)
C10—N4—C9—S1	−1.0 (13)	C6—C7—C8—C1	178.6 (10)
C9—N4—C10—C15	−7.2 (14)	C2—C7—C8—C1	−0.7 (9)
C10—N4—C9—N3	179.7 (7)	N4—C10—C11—C12	−176.1 (7)
C9—N4—C10—C11	170.5 (8)	C15—C10—C11—C12	1.6 (13)
O1—C1—C8—N2	−2.3 (14)	N4—C10—C15—C14	177.9 (8)
O1—C1—C8—C7	180.0 (8)	C11—C10—C15—C14	0.3 (14)
N1—C1—C8—N2	178.5 (8)	C10—C11—C12—I1	173.5 (6)
N1—C1—C8—C7	0.8 (9)	C10—C11—C12—C13	−2.7 (13)
N1—C2—C3—C4	179.0 (9)	I1—C12—C13—C14	−174.5 (7)
C7—C2—C3—C4	0.8 (14)	C11—C12—C13—C14	1.7 (13)
N1—C2—C7—C6	−179.0 (8)	C12—C13—C14—C15	0.3 (14)
N1—C2—C7—C8	0.4 (9)	C13—C14—C15—C10	−1.3 (14)

Symmetry codes: (i) *x*+1, −*y*+1/2, *z*−1/2; (ii) *x*, −*y*+1/2, *z*−1/2; (iii) −*x*, *y*+1/2, −*z*+1/2; (iv) *x*+1, *y*, *z*; (v) *x*−1, −*y*+1/2, *z*+1/2; (vi) *x*, −*y*+1/2, *z*+1/2; (vii) *x*−1, *y*, *z*; (viii) −*x*, −*y*, −*z*+1; (ix) −*x*−1, −*y*, −*z*+1; (x) −*x*+1, *y*−1/2, −*z*+1/2; (xi) *x*+1, −*y*+1/2, *z*+1/2; (xii) −*x*, *y*−1/2, −*z*+1/2; (xiii) *x*−1, −*y*+1/2, *z*−1/2; (xiv) −*x*+1, *y*+1/2, −*z*+1/2.

### Hydrogen-bond geometry (Å, °)

**Table:**

*D*—H···*A*	*D*—H	H···*A*	*D*···*A*	*D*—H···*A*
N1—H1···O1^ix^	0.86	2.09	2.939 (9)	169
N3—H3A···O1	0.86	2.07	2.761 (9)	137
N4—H4A···N2	0.86	2.18	2.618 (9)	111
C15—H15···S1	0.93	2.51	3.183 (10)	129

Symmetry codes: (ix) −*x*−1, −*y*, −*z*+1.
